# Genetic, Structural, and Molecular Insights into the Function of Ras of Complex Proteins Domains

**DOI:** 10.1016/j.chembiol.2014.05.010

**Published:** 2014-07-17

**Authors:** Laura Civiero, Sybille Dihanich, Patrick A. Lewis, Elisa Greggio

**Affiliations:** 1 Department of Biology, University of Padova, Via U. Bassi 58/b, Padova 35131, Italy; 2 Department of Molecular Neuroscience, UCL Institute of Neurology, Queen Square, London WC1N 3BG, UK; 3 School of Pharmacy, University of Reading, Whiteknights, Reading RG6 6AP, UK; 4 Centre for Integrated Neuroscience and Neurodynamics, University of Reading, Whiteknights, Reading RG6 6AP, UK

## Abstract

Ras of complex proteins (ROC) domains were identified in 2003 as GTP binding modules in large multidomain proteins from *Dictyostelium discoideum*. Research into the function of these domains exploded with their identification in a number of proteins linked to human disease, including leucine-rich repeat kinase 2 (LRRK2) and death-associated protein kinase 1 (DAPK1) in Parkinson’s disease and cancer, respectively. This surge in research has resulted in a growing body of data revealing the role that ROC domains play in regulating protein function and signaling pathways. In this review, recent advances in the structural information available for proteins containing ROC domains, along with insights into enzymatic function and the integration of ROC domains as molecular switches in a cellular and organismal context, are explored.

## Teaser

Ras of complex protein domains are GTP binding domains found in the ROCO family of proteins. Civiero et al. summarize recent advances in our understanding of the biology of these domains and efforts to target them in human disease

## Main Text

The Ras of complex proteins (ROC) domain was first established as a distinct protein domain family following the identification of a guanosine triphosphate (GTP) binding motif in a series of large multidomain proteins in the Amoeba *Dictyostelium discoideum* (Bosgraaf and Van Haastert, 2003). Since 2003, ROCO proteins have been identified in a range of species, from prokaryotes to humans. Interest in the structure and function of ROC domains increased with the identification of links between several proteins containing ROC domains and human disease: most notably, leucine-rich repeat kinase 2 (LRRK2) with Parkinson’s disease (PD) and death-associated protein kinase 1 (DAPK1) with cancer. ROC domains likely act as molecular switches, controlling function and, through this, the cellular role of the proteins within which they reside. This has led to analogies being drawn between ROC domains and the function of small GTPases such as Ras, as well as G protein α subunits. Over the past five years, our understanding of these proteins has been much improved by structural studies, in vitro and cellular analysis of function, and in vivo modeling. What emerges from these data is an incomplete but tantalizing picture of ROC domain function, the highly complicated mechanisms by which these domains are regulated, and the pathways that they control.

### Evolutionary and Genetic Perspective

Proteins containing ROC domains have been recognized and studied for almost two decades (Deiss et al., 1995); however, the first formal description of the ROCO protein family dates from 2003 (Bosgraaf and Van Haastert, 2003). This family comprises proteins with ROC, invariably followed by a domain termed COR (C-terminal of ROC). Phylogenetic analysis of different ROC domains revealed a monophyletic group distinct from the rest of the GTPases (Bosgraaf and Van Haastert, 2003). There are no clear examples of ROC or COR domains occurring in isolation, suggesting that the ROC-COR is likely to be a single functional unit. Another unique property of ROC-COR is that it always sits in multidomain proteins, from the simplest arrangement observed for animal MFHAS1 or plant Tornado proteins where ROC-COR is N-terminally preceded by leucine-rich repeats (LRRs) to the complex multidomain GbpC protein of *D. discoideum* that contains nine domains (Marín et al., 2008).

Although ROC domains have garnered considerable interest, their biological function is still poorly understood. From an evolutionary perspective, ROC domains are present among the most ancient and simple living organisms, including bacteria. Prokaryotic ROC domains are part of multidomain proteins that typically possess N-terminal LRRs and a C-terminal ROC-COR unit. The best-characterized bacterial ROC domain is found in the thermophilic green sulfur bacteria *Chlorobium tepidum*. A similar architecture is also present in other gram positive bacteria such as purple bacteria and cyanobacteria. Archaea also possess ROC domains with a similar architecture (Doolittle, 2000). However, blastp searches using *C. tepidum* (bacteria) or *M. Barkeri* (archaea) COR domains against myxobacteria sequences reveal no significant domain conservation. Although it is unclear whether ROC domains originated in prokaryotes or were instead horizontally transferred from eukaryotes, their presence in both archaea and bacteria suggest an ancient origin (Marín et al., 2008). The Amoebozoan slim mold *D. discoideum* possesses 11 ROCO genes, which are thought to have evolved recently from prokaryotic ROCO genes (Marín et al., 2008). *D. discoideum* ROCO genes have been extensively studied, revealing their involvement in chemotaxis and also in cell division and development through control of cytoskeleton dynamics (van Egmond and van Haastert, 2010). The *D. discoideum* ROCO gene GbpC regulates cytoskeleton assembly by cGMP-dependent phosphorylation of myosin II upon cAMP stimulation (Kortholt et al., 2012; van Egmond et al., 2008). Lack of ROCO genes in fungi and yeasts, where chemotaxis (movement of cells in response to external chemical stimuli) is less important for their life cycle compared to slime molds (Arkowitz, 1999), suggests that these genes play a role in cytoskeleton-related processes culminating in cellular or subcellular movements.

A recent bioinformatics analysis of myotubularin genes in eukaryotes identified a novel group containing ROC domains in another Amoebozoan, *Entamoeba histolytica* (Kerk and Moorhead, 2010). Myotubularins belong to the tyrosine phosphatase family and act as lipid phosphatases cleaving the D3 phosphate from phosphatidylinositol phospholipids (Schaletzky et al., 2003). These phospholipids localize to intracellular membranes and to plasma membrane microdomains and rafts, and they are thought to mediate vesicular trafficking, the transition between endosomes and lysosomes, retromer transport, and endocytosis in a phosphorylation-dependent manner (Clague and Lorenzo, 2005). Kerk and Moorhead found a large set of 19 myotubularin genes in *E. histolytica*, 9 of which contain inactive myotubularin at the N terminus followed by LRRs, ROC-COR, and kinase domains. They called this novel architecture IMLRK (inactive myotubularin-LRRs-ROCO-kinase) (Kerk and Moorhead, 2010). Compared to *D. discoideum*, the life cycle of *E. histolytica* is simpler; however, complex membrane remodeling processes linked to invasive contact with host tissue likely require a large collection of myotubularins to be finely regulated in time and space. Interestingly, extensive data support a role for human LRRK2 in vesicular trafficking (Beilina et al., 2014; Piccoli et al., 2011; Shin et al., 2008) lipid raft association (Hatano et al., 2007), and exosome formation (Fraser et al., 2013), supporting a role for ROC domains in lipid remodeling.

**Figure 1:**
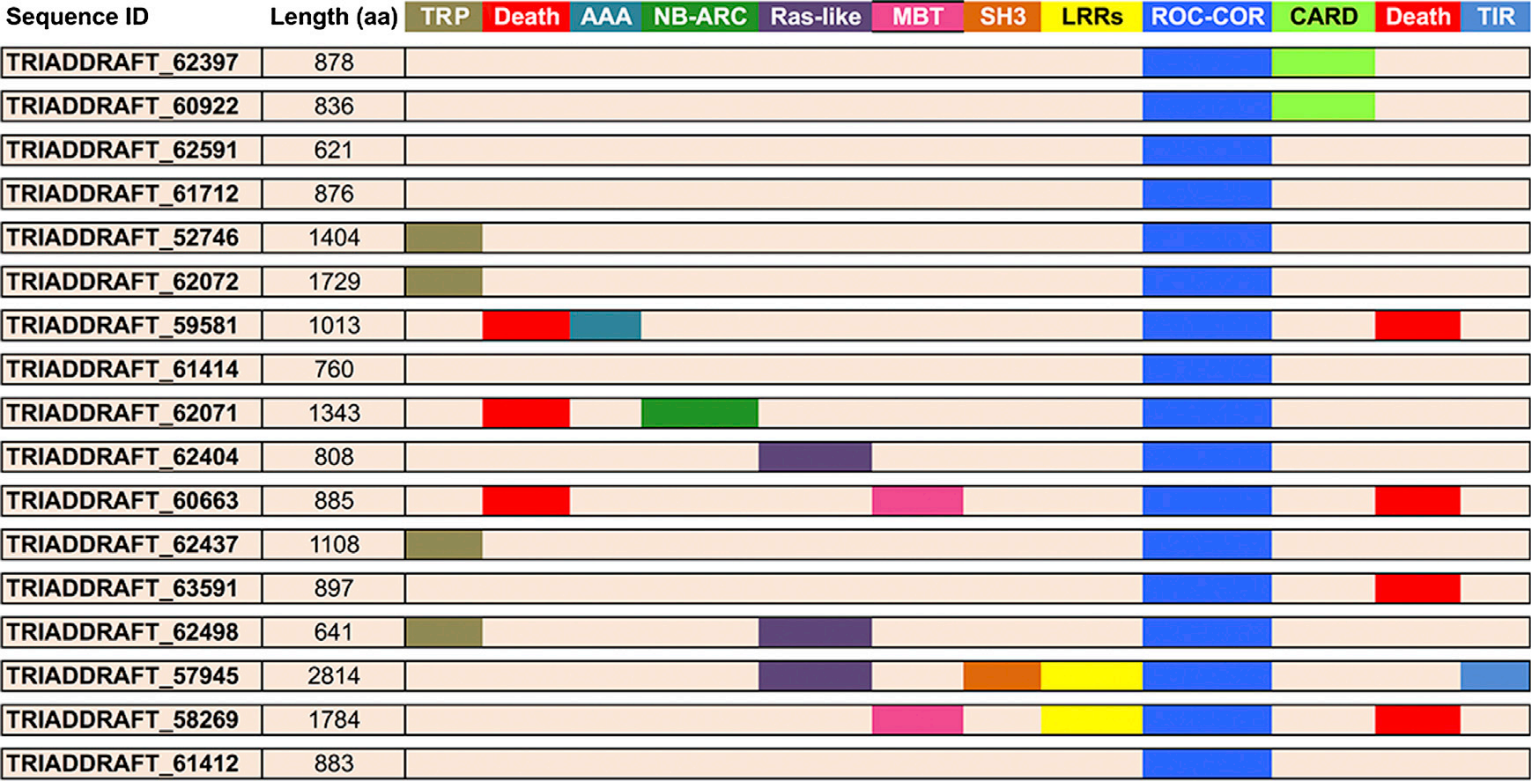
Putative Proteins Containing ROC-COR Domains from *T. adherens* Proteins were identified using blastp searches with *D. discoideum* COR domains against *T. adherens* genome (TAXID: 10228). TRP, tetratricopeptide domain; AAA, ATPase domain; MBT, malignant brain tumor repeats; SH3, SRC homology 3 domain; LRRs, leucine-rich repeats; CARD, caspase recruitment domain; TIR, Toll-interleukin receptor domain.

A bioinformatic analysis of ROC domains in *Trichoplax adhaerens*, the only species of the phylum placozoa and the most ancient metazoan known, reveals the presence of multiple ROCO genes. *T. adherens* is a simple, disc-shaped organism with two epithelial layers covering an inner layer of fiber cells and no apparent nerve, muscle, or sensory cells (Srivastava et al., 2008). Using multiple blastp searches, at least 17 putative ROCO genes can be identified in *T. adherens* (Figure 1). As shown in Figure 1, all putative protein products contain an ROC-COR domain surrounded by other functional domains, including CARD and death domains, tetratricopeptide (TRP) and LRRs repeats, and ATPase domains of the AAA family. Of interest, at least three putative protein products are predicted to contain Ras-like domains N-terminally of ROC (TRIADDRAFT_62404, TRIADDRAFT_62498, and TRIADDRAFT_57945). This arrangement is novel and particularly intriguing as two GTP binding and/or GTPase domains are present in the same protein, and it may support a model where the ROC-COR unit functions as a nucleotide-dependent dimerization device while the Ras-like GTPase acts as the signaling output (analogous to kinase domains found in some ROCO proteins). Whether this model is correct and can be extended to other ROCO proteins remains to be investigated. Another point to consider is why two divergent species such as slime molds and placozoa have independently undergone multiple gene duplication events to expand their set of proteins with ROC domains. Functionally, both organisms move in response to chemoattractants and feed by phagocytosis (Srivastava et al., 2008; van Egmond and van Haastert, 2010). Based on the established role of *D. discoideum* GbpC protein in chemotaxis and the role of human ROCO proteins in processes related to phagocytosis in response to host infection (MASL1 and LRRK2), it can be speculated that slime molds and placozoa have independently acquired multiple ROCO genes in a process of convergent evolution.

There are other examples of organisms possessing multiple ROCO genes. Zambounis et al. identified 37 LRR-GTPases of the ROCO family in the brown algae *Ectocarpus siliculosus* by using bioinformatic searches (Zambounis et al., 2012). The majority of *Ectocarpus* ROCO proteins have N-terminal LRRs followed by an ROC-COR domain and a C-terminal domain homologous either to other ROCO proteins or to transmembrane proteins. The authors found that the majority of ROCO loci are organized in clusters and that the LRR of all ROCO proteins (with one exception) exhibit a repetitive intro-exon structure where each LRR is encoded by a 72-nucletide/24-amino-acid-long individual exon also present in noncoding regions. This striking arrangement suggesting highly dynamic exon shuffling, together with the remarkable expansion of the *Ectocarpus* ROCO family, hints that *Ectocarpus* ROCO proteins may be involved in immune response mechanisms. A role of ROC domains in immune response mechanisms is gaining attention among human ROCO proteins; for example, MASL1 and LRRK2 have been shown to be upregulated upon pathogen infection (Gardet et al., 2010; Ng et al., 2011). Although the molecular mechanisms through which human ROCO proteins modulate inflammatory response are still unclear, the LRRs of LRRK2 display a significant similarity to those found in NOD-2 (Hakimi et al., 2011), an intracellular recognition receptor, suggesting that LRRK2, and possibly MASL1, may function as cytoplasmic receptors initiating NF-kB signaling in response to various danger signals and pathogen-associated molecular patterns.

As discussed, cyanobacteria possess ROC domains, but this is not true for all photosynthetic organisms. Bioinformatic searches for conserved ROC-COR domains in green algae, ferns, gymnosperms, and angiosperms reveal that only flowering plants possess ROCO genes, named Tornado1 proteins. These proteins possess N-terminal LRRs, a ribonuclease-inhibitor-like subfamily, and a C-terminal ROC-COR. Knockout (KO) studies of *Arabidopsis thaliana TORNADO1* gene revealed that Tornado1, together with Tornado2 (a tetraspanin protein), is involved in leaf patterning processes including leaf symmetry and venation patterning (Cnops et al., 2006). Due to the limited number of studies on Tornado1 proteins in plants, their function is poorly understood, as is the role of ROC domains in the signaling processes mediated by these proteins.

**Figure 2:**
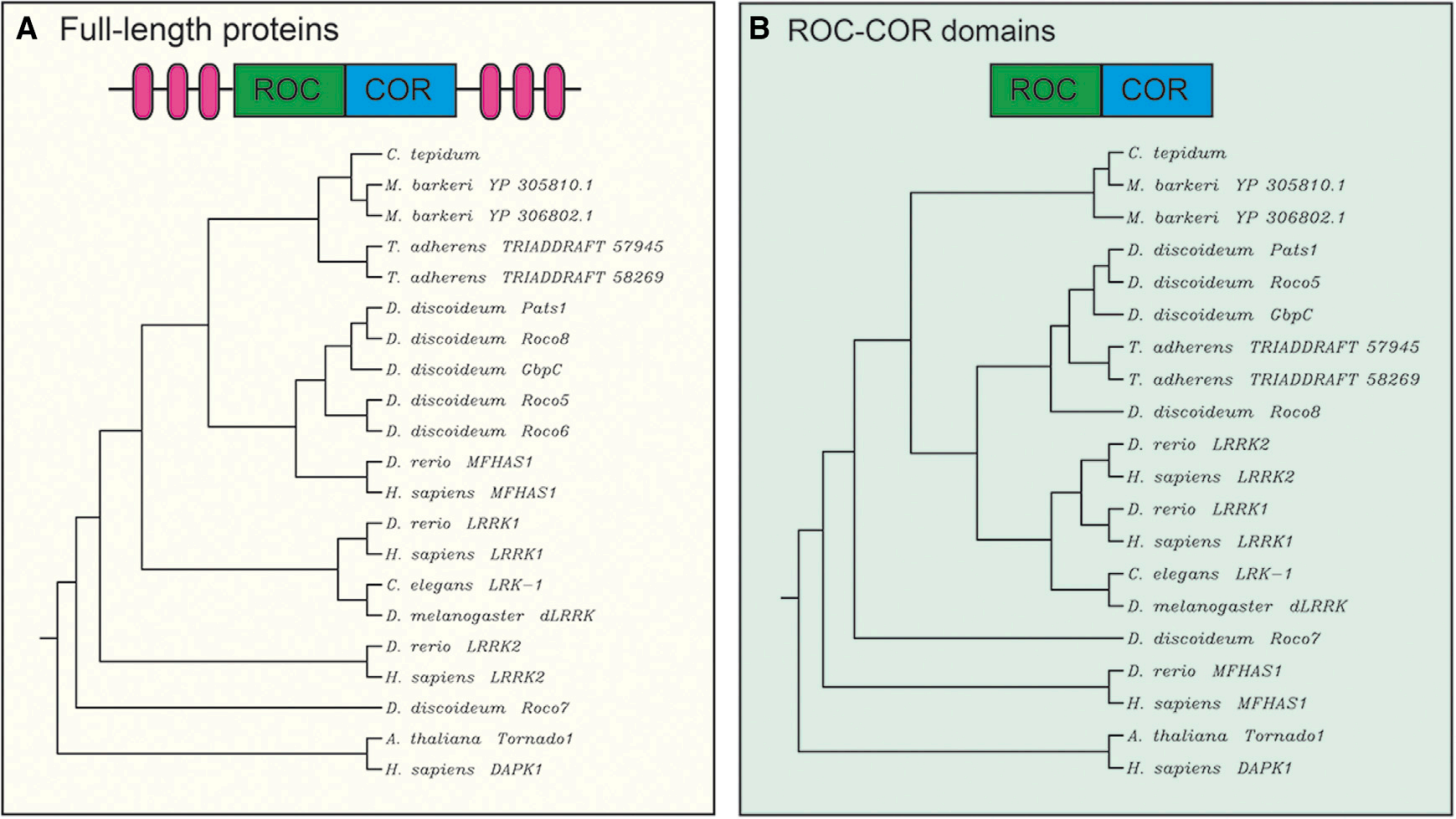
Phylogenetic Relationships among ROCO Proteins (A) Phylogenetic dendograms (constructed using the unweighted pair-group method of analysis) based on the full-length amino acid sequences of prokaryotes (*C. tepidum*), archea (*M. barkeri*), placozoa (*T. adherens*), slime mold (*D. discoideum*), plants (*A. thaliana*), invertebrates (*C. elegans* and *D. melanogaster*), and vertebrates (*D. rerio* and *H. sapiens*). Of note, the closest homolog of human DAPK1 is plant Tornado1; MFHAS1 proteins are closer to *Dyctiostelium* ROCO than to LRRKs; and *Drosophila* LRRK and *Caenorabditis* LRK-1 are closer to LRRK1 than to LRRK2. (B) Phylogenetic dendograms from the same species using the predicted ROC-COR domains. LRRK1’s closest ROC-COR domain is LRRK2.

The evolutionary history of ROCO genes in animals has been thoroughly reconstructed by Marin (Marín et al., 2008). Protostomes and deuterostomes possess LRRK and DAPK1 genes, while only deuterostomes have MFHAS1 genes. The phylogenetic relationships among prokaryotes, archea, placozoa, slime molds, plants, invertebrates, and vertebrates ROCO proteins are shown in Figure 2.

Several groups have reported evidence for multiple splice variants of the *LRRK2* gene. A study by Giesert et al. examined splicing of *LRRK2* in the mouse brain, uncovering evidence of altered splicing of exon 5 and a novel exon 42 (located within the kinase domain of this protein) (Giesert et al., 2013). Of direct relevance to ROC domain function, Trabzuni and colleagues reported that LRRK2 may undergo alternative splicing events around exons 32 and 33 in the substantia nigra (Trabzuni et al., 2013). Although these observations are limited to the RNA level and need to be confirmed by demonstrating the existence of the corresponding protein isoforms, it is noteworthy that this nucleotide region corresponds to the ROC-COR domain of LRRK2. It could be speculated that these substantia-nigra-specific LRRK2 isoforms lacking part of the ROC-COR may play a pathological function by acting, for instance, as dominant negative. It is likely that these reports represent the tip of the iceberg with regard to splicing of ROC domain containing genes, and much more remains to be uncovered regarding all of the ROCO proteins.

### Functional Conservation of ROC Domain Activity

With regard to the biological function of the ROC domain, guanosine nucleotide binding and hydrolysis have been demonstrated for several ROCO proteins. Guanosine nucleotide binding has been reported for the four human ROCO proteins (Carlessi et al., 2011; Dihanich et al., 2014; Ito et al., 2007; Jebelli et al., 2012; Korr et al., 2006). Whether the ability to bind GTP is conserved in other organisms has been tested in two cases—for the *D. discoideum* ROCO protein GbpC (van Egmond et al., 2008) and for the *C. tepidum* ROCO protein (Gotthardt et al., 2008). Based upon sequence homology and extant functional data, it is likely that ROC domains where key catalytic residues are conserved are able to bind nucleotides.

The ability of ROC domains to hydrolyze GTP has been investigated for a number of ROCO proteins. Several groups have demonstrated that LRRK2 is able to bind and hydrolyze GTP (Guo et al., 2007; Lewis et al., 2007; Li et al., 2007). DAPK1 has also been reported to possess GTPase activity (Carlessi et al., 2011).

An important aspect of ROC domain biology is the impact of guanosine nucleotide binding on the structure and function of neighboring domains. The majority of ROCO proteins have functional domains (including enzymatic activities) in addition to their ROC domains. An early observation was that manipulating the GTP binding properties of LRRK1 had a major impact on the kinase activity of this protein, leading the authors to propose a model for LRRK1 function, and by implication the ROCO proteins (Korr et al., 2006). In this model, the cycle between GTP-bound and GDP-bound ROC controls the kinase activity of LRRK1 in a manner analogous to the control of Raf kinase activity by the Ras proteins. These data were supported by studies investigating LRRK2, with artificial mutations excluding guanosine nucleotides reducing kinase activity (Ito et al., 2007). More recent data suggest that the kinase activity of this protein is dependent upon whether a guanosine nucleotide of any type is bound to the ROC domain (Taymans et al., 2011). This is consistent with a model proposed by Gasper and coworkers, suggesting that ROC domains act in a similar fashion to G proteins, dimerizing upon GTP binding (Gasper et al., 2009). An insight into the relationship between ROC domains and the other functions of the ROCO proteins is provided by two recent reports investigating ROC domain function in DAPK1 (Carlessi et al., 2011; Jebelli et al., 2012). The kinase domain of DAPK1 sits at the extreme N terminus of the protein, in contrast to LRRK1 and LRRK2 where the kinase domain sits in the C terminus of the protein. When guanosine nucleotide binding is disrupted by artificial mutations in DAPK1, kinase activity does not decrease. This clear divergence from the biology of LRRK2 suggests that the role of ROC domains in controlling other enzymatic functions is complicated and is likely to be dictated by the 3D organization of these domains.

**Figure 3:**
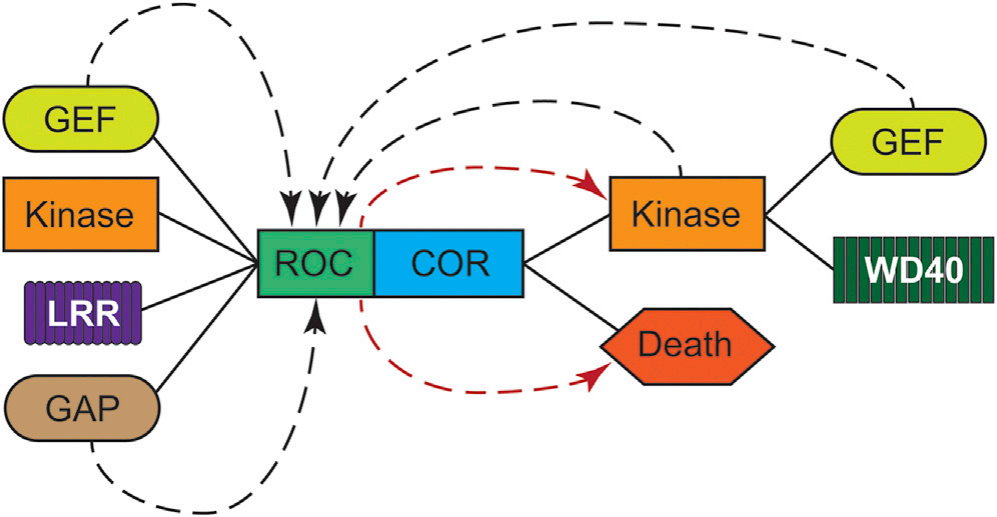
Functional Interactions between the ROC Domain and Other Protein Domains Associated with It

A final aspect of the relationship between the ROC domain and its flanking enzymatic activities or regulatory domains is the reciprocity of these relationships. Data from LRRK2 have highlighted the presence of a number of autophosphorylation sites within the ROC domain of this protein. While the physiological role of these phosphorylation events remains unclear, their identification suggests that a complex pattern of regulation exists between the different enzymatic activities of the ROCO proteins. Phosphorylation of the ROC domain may act to regulate guanosine nucleotide binding (Webber et al., 2011). One caveat is that mutation of individual autophosphorylation sites may have a structural rather than functional impact, as suggested by the fact that kinase-inactive mutants are competent in binding and hydrolyzing GTP (Biosa et al., 2013). The possible interactions between the ROC-COR domain and surrounding domains are summarized in Figure 3. This area of ROC biology bears greater scrutiny: for example, the *D. discoideum* ROCO protein Gbpc, which possesses a C-terminal guanine exchange factor (GEF) domain in addition to its ROC and kinase activities. Examination of Gbpc biology suggests that this GEF domain interacts with and regulates the ROC activity of this protein, adding further complexity (van Egmond et al., 2008).

### Structural Perspective

The production of highly pure, full-length recombinant ROCO proteins for structural studies is challenging. To date, no full-length ROCO protein structures have been solved, with limited data available for the ROC-COR or ROC alone (Deng et al., 2008; Gotthardt et al., 2008; Liao et al., 2014). Published in 2008, the first structure of the ROC domain from the human ROCO protein LRRK2 revealed a dimeric GTPase (Deng et al., 2008). Although the proposed model describes a canonical GTPase fold, the catalytic core of LRRK2-ROC adopts an unusual topology because of domain swapping, in which the N-terminal part of one domain interacts with the C-terminal one of the other. In the same year, a crystallographic study of the ROC-COR unit from the bacteria *C. tepidum* was published confirming the dimeric organization of the ROCO proteins. In contrast to the previously determined structure of the human ROC domain, the structural analysis revealed a canonical G protein domain where dimerization is mediated by the C-terminal half of the COR domain and by highly conserved residues on the ROC-ROC interface (Gotthardt et al., 2008). More recently, Liao and coworkers derived a monomeric model structure for the human ROC domain (Liao et al., 2014). While the nucleotide free form of this protein formed a mixture of the monomer and dimeric complex, GDP or GppNHp binding caused the ROC domain to adopt a monomeric conformation, potentially consistent with data investigating the kinase activity of LRRK2 and the G protein activated by the nucleotide-dependent dimerization (GAD) model for ROCO function. Studies of isolated ROC domains are obviously limited with regard to the quaternary structure of ROCO proteins. As described for dynamins, other domains may be required for dimerization and/or oligomerization.

Although high-resolution data for ROCO protein tertiary structures are limited, it is probably that LRRK2 and other members of the ROCO family are functional dimers (Berger et al., 2010; Greggio et al., 2008; Jebelli et al., 2012; Klein et al., 2009; Sen et al., 2009). The hypothesis of a homodimeric or heterodimeric conformation by the ROCO family is also supported by a growing body of literature based on size exclusion chromatography assays and immunogold labeling transmission electron microscopy analysis of full-length purified proteins (Carlessi et al., 2011; Civiero et al., 2012; Dihanich et al., 2014; Greggio et al., 2008; Jebelli et al., 2012).

Due to their low affinity (in the range of μM) for nucleotides (Civiero et al., 2012; Gotthardt et al., 2008) and to their capability to dimerize or oligomerize (Civiero et al., 2012; Sen et al., 2009), the ROCO proteins were recently suggested to act as GADs, a category including dynamin and septins (Gasper et al., 2009). GADs are a group of proteins that do not require GEFs to exchange GDP for GTP (Gasper et al., 2009). The GTP-bound dimer is the active form that is responsible for the biological process, which is terminated by hydrolysis of GTP. Supporting the hypothesis of ROCO proteins as functional dimers, it was shown that the ROC-COR module from *C. tepidum* depends on a dimeric conformation to hydrolize GTP, with mutations analogous to the ROC-COR Parkinson disease mutations (R1441C, Y1699C, I1371V) located in the ROC-COR interface leading to a reduction of GTPase activity (Gotthardt et al., 2008). In addition, human LRRK2 purified proteins carrying R1441C or Y1699C mutations that show a disrupted GTPase activity (Daniëls et al., 2011; Lewis et al., 2007) bind the ROC domain with less affinity in vitro compared to the wild-type protein (Li et al., 2009a).

### Cellular and Organismal Function

The ROCO proteins have been implicated in a range of cellular processes. In slime molds, the key phenotype with which they are linked is chemotaxis (Bosgraaf et al., 2002). KO of GbpC and Pats1 modulate chemotaxis and cytokinesis, respectively (Bosgraaf et al., 2005; Abysalh et al., 2003). Studies of both GbpC and Pats1 highlight one of the major confounding issues in studying ROC domain biology: separating out whether a given phenotype is associated with the activity of the ROC domain of a protein or dependent upon the function of the holoprotein in toto. Whole gene KO represents a robust method to examine function; however, elucidating the contribution of individual domains to KO phenotypes is not straightforward. In the ROCO proteins, this is complicated by the interactions between multiple enzymatic activities. In *D. discoideum* studies have been carried out on GbpC to address this issue, investigating the contribution of the various domains of GbpC to the chemotaxis phenotype (van Egmond et al., 2008). More recently, Roco4, the *D. discoideum* protein most closely related to LRRK2, has been studied as a system for modeling the impact of mutations in LRRK2 (Gilsbach et al., 2012). Ablation of Roco4 results in an inability to synthesize cellulose under starvation conditions, preventing the formation of functional fruiting bodies. Finally, the ROCO kinase QkgA has been implicated in chemotaxis and cell proliferation although the precise mechanisms regulating these links have not yet been defined (Phillips and Gomer, 2010, 2012).

Research using more complex organisms has implicated ROCO proteins in a number of cellular phenotypes. Knockout of LRK-1, the *C. elegans* LRRK ortholog, is associated with altered polarized sorting of synaptic vesicles (Sakaguchi-Nakashima et al., 2007). Using a kinase dead form of LRK-1, the authors report a kinase dependency of this phenotype; however, the role of the ROC domain of LRK-1 has not been directly examined. A study by Sämann and coworkers examined LRK-1 in the context of stress response and neurite outgrowth, an area of great interest with regard to LRRK2 in human models (see below), with their results suggesting that LRK-1 is involved in the response to endoplasmic reticulum stress caused by exposure to tunicamycin (Sämann et al., 2009). Subsequent studies have used *C. elegans* as a system to examine the function of human LRRK2, a number of which have examined Parkinson’s disease mutations located in the ROC domain (Saha et al., 2014; Yao et al., 2013).

*C. elegans* also possesses a DAPK1 ortholog, with several reports implicating this in the control of macroautophagy and wound closure (Chuang and Chisholm, 2014; Kang and Avery, 2010). Kang and colleagues demonstrated that DAPK1 operates downstream of the muscarinic receptors in the worm to control the autophagic response to starvation, with knockdown or knockout of this gene leading to reduced response (Kang et al., 2007). An analysis of the role of DAPK1 in wound closure in *C. elegans* revealed that it acts as a negative regulator of this process, downstream of Ca^+^ signaling (Tong et al., 2009; Xu and Chisholm, 2011).

Although the *D. melanogaster* genome does include a DAPK ortholog (encoded by the *DRAK* gene), this protein does not possess an ROC domain (Chuang and Chisholm, 2014). In contrast, the *Drosophila* LRRK ortholog (dLRRK2) has been the subject of detailed investigations, with knockout and targeted mutation models developed. An observation directly relevant to the biology of the ROC domain is that *dLRRK* KO results in a neurodegenerative phenotype (Lee et al., 2007). However, if just the kinase domain is removed (and the ROC domain remains), then there is no neuronal cell death (Wang et al., 2008). This suggests that the cellular triggers leading to cell death following the loss of dLRRK depend upon the activity of the ROC domain. Several studies have used *Drosophila* as a model system to examine pathways linked to LRRK2 biology—for example, by identifying 4EBP1 as a putative substrate (Imai et al., 2008), implicating LRRK2 in microRNA regulation of translation (Gehrke et al., 2010), and suggesting that LRRK2 is involved in membrane fusion involving the endophilin proteins (Matta et al., 2012).

There are numerous rodent models for ROCO protein function. Both DAPK1 and LRRK1 KO mice have been developed. The former have no obvious gross phenotype; however, Tu and colleagues used the model to examine a role for DAPK1 in NMDA mediated brain damage following a ischemic stroke (Tu et al., 2010). KO of LRRK1 results in osteopetrosis, although the altered pathways leading to this are unclear (Xing et al., 2013). KO of LRRK2 results in disruption of kidney, lung, and liver function, including the accumulation of vesicles and α-synuclein within cells in these tissues and alterations in markers for autophagy (Tong et al., 2010, 2012). Data from subsequent studies suggest that this may be a kinase-dependent phenotype (Herzig et al., 2011), and it is reproduced in rats lacking LRRK2 (Baptista et al., 2013). LRRK2 KO mice have also been reported to display increased susceptibility to an experimentally induced form of inflammatory bowel disease, possibly due to altered nuclear factor of activated T cells (NFAT) transcriptional regulation (Liu et al., 2011). A number of transgenic and knockin mouse models for LRRK2 display some neuronal phenotypes, with marked tau pathology a characteristic of a bacterial artificial chromosome transgenic mouse with a mutation in the ROC (the R1441G mutation) (Li et al., 2009b).

The cellular functions of the four human ROCO proteins have been the subject of intense scrutiny. Several reports have linked the cellular function of LRRK1 to endosomal sorting, in particular to trafficking of the epidermal growth factor receptor (Hanafusa et al., 2011; Ishikawa et al., 2012). Human genetics has linked LRRK2 to Parkinson’s disease, Crohn’s disease, multibacillary leprosy, and cancer (Lewis and Manzoni, 2012). While the molecular mechanisms underpinning these associations are unclear, there are a number of common themes that emerge. These include inflammation, the immune system, and cell fate. How the ROC domain contributes to these disease phenotypes is not clear, although the location of mutations (for example, the R1441C mutation) in the ROC domain causative for Parkinson’s disease has focused a great deal of research on this aspect of LRRK2 biology. At a cellular level, LRRK2 has been implicated in a wide range of cell processes including mitochondrial biology, synaptic vesicle cycling, macroautophagy, cytoskeletal dynamics, and the control of translation (Cookson, 2010). A recurring observation is an association of LRRK2 with membranes, including mitochondria, autophagosomes, and synaptic vesicles (Alegre-Abarrategui et al., 2009; Berger et al., 2010; Biskup et al., 2006). LRRK2 has been linked to the Rab GTPases, involved in the regulation of intracellular membrane fusion events (Beilina et al., 2014; MacLeod et al., 2013). Given the localization of LRRK2 to membranous structures, it is possible that the ROC domain may fulfil a similar role to the Rabs, despite the sequence divergence between these domains. Insights into the biological function of the LRRK2 ROC domain are provided by experiments studying PD mutations in the ROC and kinase domain. Several of these investigations report a divergence between the impact of mutations in the ROC and the kinase domains. These data—for example, the differential impact of the R1441C and G2019S on translational phenotypes linked to LRRK2 (Gehrke et al., 2010)—suggest that the cellular function of the ROC domain may be distinct from that of the kinase domain.

DAPK1 has been implicated in cell death pathways—in particular, type II autophagic cell death (Bialik and Kimchi, 2006; Deiss et al., 1995). Several themes emerge from studies of DAPK1, including macroautophagy and the regulation of membrane vesicle biology (Inbal et al., 2002). Both autophagy and wound healing, identified as being linked to DAPK1 in *C. elegans*, have been linked to the function of the mammalian gene (Bialik and Kimchi, 2010; Kuo et al., 2006). Although the biochemistry of the ROC domain of DAPK1 has been investigated, the impact of this domain on pathways downstream of this protein has not and is likely to be a highly fruitful line of enquiry in the future.

The cellular function of MASL1 is the least understood of the human ROCO proteins. MASL1 was originally identified as a gene amplified in malignant fibrous histiocytomas, implicating it in the control of cell fate and division (Sakabe et al., 1999). The cellular studies that have been carried out since then support this implication, suggesting a role for MASL1 in the regulation of the ERK pathway to influence erythroid differentiation of CD34 (+) cells (Kumkhaek et al., 2013) and in necrotic cell death (Dihanich et al., 2014).

### ROC Domains as Pharmacological Targets

Given the important role played in human disease by proteins containing ROC domains, it is perhaps unsurprising that ROC domains are considered as potential therapeutic targets. At present, the prevailing strategy to target LRRK2 and DAPK1 in a disease context is to modulate kinase activity. Kinase inhibitors have been developed for both LRRK2 and DAPK1 (Deng et al., 2011; Okamoto et al., 2009), with a large number of small molecule inhibitors reported for LRRK2 (Choi et al., 2012; Reith et al., 2012; Zhang et al., 2012). In contrast, there is a single published report of targeting ROC biology, investigating both LRRK2 and DAPK1 (Klein et al., 2009). Klein and coworkers expressed the ROC domain of LRRK2 as a transgene alongside full-length LRRK2 and observed an inhibition of LRRK2 activity. This echoes an earlier report targeting the *Dictyostelium* protein Pats1 (Abysalh et al., 2003). In this study, expression of the Pats1 ROC domain was able to exert a dominant negative effect on downstream cytokinetic pathways, suggesting that the interruption of complex formation acts to inhibit function. These data have a number of implications for ROC domain biology; however, the transgenic approaches required to translate these findings into a viable in vivo therapeutic strategy are not yet amenable for application in a clinical setting.

**Figure 4:**
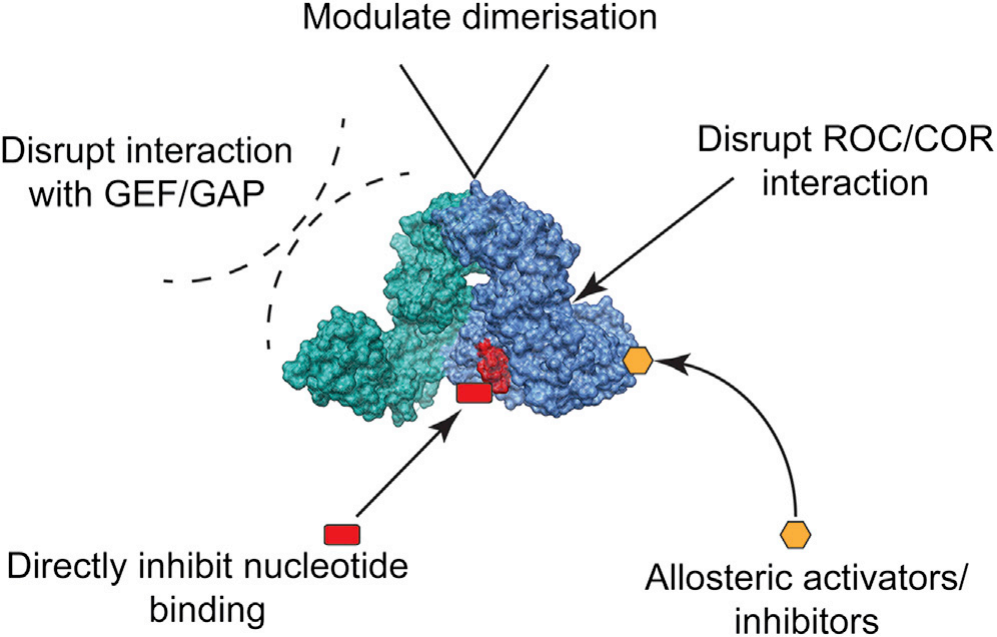
Strategies to Target ROC Domain Activity Image of *C. tepidum* ROCO protein derived from Protein Data Bank reference 3PDU (Gotthardt et al., 2008).

As our understanding of the function of the ROC domain increases, so do opportunities to target its biology and pathological consequences (Figure 4). In particular, there is the potential to benefit from previous attempts to target the activity of GTPases. Two case studies are instructive: that of Ras in human cancer and that of G protein α subunits in G protein coupled receptor-signaling pathways.

Following the identification of the Ras genes (H*-Ras*, K-*Ras*, and N-*Ras*), and the close association between point mutations in these genes and human cancer, substantial efforts have been made to correct their oncogenic activity (Karnoub and Weinberg, 2008). As it became clear that the biochemical fault linking these proteins to tumor formation was a reduction in GTPase activity, altering the downstream effects of this become a priority target for the cancer research field. Although a great deal is now known about the structural basis for GTP hydrolysis by Ras proteins, efforts to target this pathway have focused on the downstream effectors of Ras, intervening in the interactions between Ras proteins and these effectors, altering the binding of proteins directly regulating GTP hydrolysis or guanine exchange factors, or by manipulating the processing and cellular location of Ras proteins. This strategy derives from the intrinsic difficulties of altering the rate of GTP hydrolysis in a specific manner and the lack of potential small molecule binding pockets on Ras to facilitate allosteric regulation (Downward, 2003). Recent successes include using small molecules to modulate the interaction between K-Ras and Son of Sevenless (SOS), a GEF (Maurer et al., 2012), directly inhibiting the interactions between H-Ras and Raf with small molecules derived from an in silico screen (Shima et al., 2013) and disrupting the interaction between K-Ras and PDEδ, a prenyl binding protein that acts to govern the cellular localization of K-Ras (Zimmermann et al., 2013). Most recently, Ostrem and coworkers have demonstrated that pharmacological targeting of K-Ras via an allosteric approach is tractable, and they were able to demonstrate mutation-specific inhibition—a finding of obvious relevance to LRRK2 (Ostrem et al., 2013). While it is important not to underestimate the scale of the challenge, these advances provide hope for targeting ROC domain biology.

Heterotrimeric G proteins are involved in the control of a range of cellular functions, and similar to Ras the pathways within which they function have been implicated in oncogenesis. Efforts to target G protein α subunits, the subunit of the heterotrimeric G protein complex responsible for the binding and hydrolysis of GTP, have resulted in the derivation of a number of compounds that modulate the release and exchange of guanosine nucleotides. Examples include suramin (Butler et al., 1988) and imidazopyrazines (Ayoub et al., 2009), molecules that are thought to inhibit nucleotide exchange (Smrcka, 2013). YM-254890 operates via a similar mechanism and has been cocrystalized with Gα_q_ (Nishimura et al., 2010). This provides a molecular insight into the mechanism of action, suggesting that YM-254890 restricts the freedom of movement for the subunit and inhibits the release of GDP from the active site.

What is revealing about the experience of targeting G proteins and Ras is that advances have been achieved not by directly targeting the active site of these proteins but by acting on protein-protein interactions or regulatory mechanisms. For the ROCO proteins, this is hindered by the lack of validated interacting proteins that modulate guanosine nucleotide hydrolysis or exchange. Candidate GAPs and/or GEFs have been identified for LRRK2; however, the spatial details of these relationships remain obscure (Biosa et al., 2013; Haebig et al., 2010). Furthermore, due to the low affinity of ROCO proteins for guanine nucleotides (in the μM range), it is still controversial whether ROCO require GEFs for nucleotide exchange. Therefore, robust validation of authentic in vivo GEFs and/or GAPs for ROCO proteins is needed before considering them as potential targets. More optimistically, the multidomain proteins containing ROC domains benefit from having a number of putative protein-protein interaction candidates within the same open reading frame. Indeed, what structural data exist for proteins containing ROC domains suggest that these interactions are critical for function. It is also notable that the sequence divergence between ROC domains and the small GTPases suggests that it may be possible to design molecules that will interact specifically with ROCO proteins. On a cautionary note, there are still major gaps in our understanding of the consequences of inhibiting or potentiating signaling pathways regulated by ROC domain GTP and GDP binding. Even for those proteins studied most intensely, DAPK1 and LRRK2, we do not have a validated model for the interactions between the enzymatic activities of these proteins. Given the important boost to research provided by the availability of specific kinase inhibitor tools for these proteins, it is likely that the development of tool compounds specifically targeting ROC domain function will prove invaluable for delineating regulatory mechanisms centered on this domain.

### Conclusions

From a standing start in 2002, our understanding of ROC domain structure and function has advanced at a startling rate, primarily driven by the realization of the role that ROC-domain-containing proteins play in human disease. It is likely that the coming years will provide even greater insights into the function of this domain, in particular with higher resolution structural data in the context of multidomain fragments of ROCO proteins. The urgent requirement for novel therapies to treat the disorders linked to proteins containing ROC domains highlights the need to examine the feasibility of targeting ROC biology, despite the huge challenge that this represents, and this is sure to be a major focus of research into the proteins in the future.
